# Acute Down-regulation of BDNF Signaling Does Not Replicate Exacerbated Amyloid-β Levels and Cognitive Impairment Induced by Cholinergic Basal Forebrain Lesion

**DOI:** 10.3389/fnmol.2018.00051

**Published:** 2018-02-22

**Authors:** Marion T. Turnbull, Zoran Boskovic, Elizabeth J. Coulson

**Affiliations:** ^1^Clem Jones Centre for Ageing Dementia Research, Queensland Brain Institute, The University of Queensland, Brisbane, QLD, Australia; ^2^Faculty of Medicine, School of Biomedical Sciences, The University of Queensland, Brisbane, QLD, Australia

**Keywords:** Alzheimer’s disease, basal forebrain, cholinergic neuron, amyloid-β, brain-derived neurotrophic factor, APP/PS1 transgenic mouse

## Abstract

Degeneration of basal forebrain cholinergic neurons (BFCNs) precedes hippocampal degeneration and pathological amyloid-beta (Aβ) accumulation, and underpins the development of cognitive dysfunction in sporadic Alzheimer’s disease (AD). We hypothesized that degeneration of BFCNs causes a decrease in neurotrophin levels in innervated brain areas, which in turn promotes the development of Aβ pathology and cognitive impairment. Here we show that lesion of septo-hippocampal BFCNs in a pre-symptomatic transgenic amyloid AD mouse model (APP/PS1 mice) increases soluble Aβ levels in the hippocampus, and induces cognitive deficits in a spatial memory task that are not seen in either unlesioned APP/PS1 or non-transgenic littermate control mice. Furthermore, the BFCN lesion results in decreased levels of brain-derived neurotrophic factor (BDNF). However, viral knockdown of neuronal BDNF in the hippocampus of APP/PS1 mice (in the absence of BFCN loss) neither increased the level of Aβ nor caused cognitive deficits. These results suggest that the cognitive decline and Aβ pathology induced by BFCN loss occur independent of dysfunctional neuronal BDNF signaling, and may therefore be directly underpinned by reduced cholinergic neurotransmission.

## Introduction

Alzheimer’s disease (AD) is histopathologically characterized by the accumulation of two proteins: extracellular deposits of amyloid-β (Aβ) that form amyloid plaques, and intracellular inclusions of the microtubule-associated protein tau that give rise to neurofibrillary tangles (NFTs). In conjunction with this pathology, post-mortem assessment of the brains of AD patients has shown that selective degeneration of the cholinergic neurons in the basal forebrain is a significant pathological feature of the sporadic form of the disease and likely underpins aspects of cognitive decline (Mesulam, 2004; Contestabile, 2011; Schliebs and Arendt, 2011). Consistent with this, modern structural magnetic resonance imaging (MRI) and positron emission tomography (PET) imaging of amyloid have demonstrated that significant basal forebrain volume loss occurs in prodromal stages of the disease (Hall et al., 2008; Grothe et al., 2013; Kerbler et al., 2015), and that this degeneration correlates with and predicts subsequent cognitive decline, cortical Aβ burden and hippocampal degeneration (Grothe et al., 2010; Kerbler et al., 2015; Schmitz and Nathan Spreng, 2016).

Transgenic mice that model specific aspects of AD have been widely used to understand the etiology of amyloidogenesis and the development of NFTs. As early degeneration of basal forebrain cholinergic neurons (BFCNs) does not occur in AD mouse models (Perez et al., 2007), an immunotoxin that selectively targets cholinergic neurons through their expression of the p75 neurotrophin receptor (p75^NTR^), the anti-p75^NTR^ antibody-conjugated ribosome-inactivating toxin (p75-saporin), has been used to induce BFCN lesions and investigate the subsequent downstream effects. BFCN lesioning in mice that are genetically programmed to overproduce Aβ exacerbates amyloid and tau pathology and increases the associated cognitive deficits (Gil-Bea et al., 2012; Laursen et al., 2013; Ramos-Rodriguez et al., 2013; Hartig et al., 2014). However, an exacerbation in tau pathology is not seen following BFCN lesioning in tau-only transgenic mice (Turnbull and Coulson, 2017), indicating that this pathology is independent of cholinergic degeneration, and is likely driven by the induced Aβ accumulation. However, it remains unclear how reduced cholinergic innervation results in an increase in Aβ in the cortex and/or hippocampus.

We and others have reported that reduced cholinergic innervation results in decreased hippocampal brain-derived neurotrophic factor (BDNF) expression (Gil-Bea et al., 2011; Turnbull and Coulson, 2017). Furthermore, in human AD post-mortem tissue, BDNF mRNA and protein, as well as its precursor form, pro-BDNF, its receptor tropomyosin receptor kinase B (TrkB) and activation of downstream signaling molecules such as cAMP response element binding (CREB) protein, are all decreased (Phillips et al., 1991; Yamamoto-Sasaki et al., 1999; Holsinger et al., 2000; Siegel and Chauhan, 2000; Peng et al., 2005). A reduction in the serum BDNF level also correlates with an increase in the severity of dementia (Laske et al., 2006, 2011; Yasutake et al., 2006). These findings implicate reduced trophic signaling in AD pathogenesis; however whether this effect is causative or a consequence of the disease is unclear.

Reduced BDNF and nerve growth factor (NGF) signaling can induce amyloidogenic processing of amyloid precursor protein (APP), which results in the apoptotic cell death of cultured hippocampal neurons (Matrone et al., 2008). Furthermore, enhancing BDNF signaling *in vitro* can reduce Aβ production or enhance the clearance (Arancibia et al., 2008) and dephosphorylation of tau protein (Elliott et al., 2005). However, genetic down-regulation of BDNF (heterozygous gene knockout) in AD mouse models does not result in exacerbated AD-like pathology (Castello et al., 2012), although these mice harbor BDNF deficits throughout their entire lives and may develop compensatory mechanisms that overcome the effect of reduced BDNF (Rantamäki et al., 2013). Therefore the effect of acute neurotrophic dysfunction, as might occur in conjunction with BFCN denervation in an AD mouse model, is yet to be studied.

In order to test the role of impaired cholinergic and neurotrophic signaling in the development of AD, we compared the effect of lesioning BFCNs with that of an acute reduction in BDNF expression in the hippocampus.

## Materials and Methods

### Animals

All experiments were approved by the University of Queensland Animal Ethics Committee, in accordance with the Australian Code of Practice for the Care and Use of Animals for Scientific Purposes. Mice were housed on a 12 h dark/light cycle with water and standard chow available *ad libitum*.

#### APP/PS1 Transgenic Mice

The APP/PS1 mice (Jax Mice Database strain B6C3 Tg (APPswe, PSEN1dE9) 85Dbo/J; stock number #004462) express human APP695 with Swedish mutations (K595N and M596L) and mutant human presenilin 1 carrying the exon-9-deleted variant (Jankowsky et al., 2004). Hemizygous APP/PS1 transgenic males aged 5 months were used, together with age- matched non-transgenic littermate controls. Only male mice were used in these studies as gender differences in plaque deposition for this transgenic line have been reported (Burgess et al., 2006).

#### Tau Knockout Transgenic Mice

A single homozygotic male tau knockout mouse (Tau KO; Jax Mice Database strain Mapttm1 (EGFP) Klt; stock number #004779) was used as a negative control for tau expression in western blotting experiments.

#### APP/PS1 × BDNF^2lox^ Transgenic Mice

APP/PS1 mice were crossed onto BDNF^2lox^ mice (Jax Mice Database strain Bdnftm3Jae; stock number #004339) which possess loxP sites flanking exon 5 of the BDNF gene. For our experiments, BDNF^2lox^ (heterozygous) × APP/PS1 male mice were used with age-, gender- and genotype-matched littermates as controls. Hereafter we refer to APP/PS1 mice crossed onto heterozygous BDNF^2lox^ mice as APP/PS1 × BDNF^fl/wt^ mice.

### Stereotaxic Surgery

Mice were anesthetized by intraperitoneal (i.p.) injection of ketamine (100 mg/kg) and xylazine (10 mg/kg). For larger mice (>35 g) zoletil (40 mg/kg) was used instead of ketamine. To lesion BFCNs, a single infusion of murine p75-saporin (0.4 mg/ml; Advanced Targeting Systems) or control rabbit IgG-saporin (0.4 mg/ml) was stereotaxically injected into the basal forebrain. The needle was lowered into the medial septum (A-P 0.9 mm; M-L 0 mm; D-V 4.2 mm from Bregma) and the toxin was infused at a rate of 0.4 μl/min (1.5 μl total volume).

Alternatively, to knock down BDNF gene expression in hippocampal neurons, a single infusion of AAV-Synapsin-Cre-GFP (SignaGen Laboratories; Serotype 8; 1 × 10^13^ genome copies/ml; SignaGen Laboratories) was stereotaxically injected into each hippocampus (A-P −2 mm; M-L ±1.3 mm; D-V 1.8 mm), with the virus infused at a rate of 0.4 μl/min (2 μl total volume). Immediately after surgery and 24 h post-surgery, mice were injected subcutaneously with the analgesic Torbugesic (2 mg/kg), and the antibiotic Baytril (5 mg/kg).

Two groups of mice underwent stereotaxic injection of AAV-Synapsin-Cre-GFP into the hippocampus, one group at 5 months of age and another at 7 months of age. All mice were analyzed 2 months post-viral infusion for BDNF protein and total Aβ levels, hippocampal amyloid plaque load, and behavioral parameters in a Y maze test. As no differences in any parameter were found between the mice of different ages, all data presented in the results are from pooled groups.

### Tissue Preparation

Mice were sacrificed either by sodium pentobarbital (100 mg/kg i.p.) overdose followed by transcardial perfusion of 4% paraformaldehyde (PFA) or by cervical dislocation. Tissue that was to be assessed by biochemical methods was dissected out from the freshly isolated brain and snap frozen in liquid nitrogen. The entire hippocampus of BFCN-lesioned mice was used for biochemical analysis, whereas for BDNF^fl/wt^ mice, only the anterior hippocampus containing the AAV-infected GFP-positive tissue was used. Brain areas to be assessed histologically were post-fixed in 4% PFA overnight at 4°C, and then left for 24 h in phosphate-buffered saline (PBS) containing 30% sucrose. The following day, the tissue was embedded in Frozen Section Compound (FSC22; Leica) and basal forebrain and hippocampal sections were cut in the coronal plane (40 μm) using a sliding microtome (SM2000r, Leica). All sections used for comparative analysis were processed, stained and analyzed together.

### Immunohistochemistry

For visualization of cholinergic neurons in the basal forebrain, free-floating sections were immunostained using an goat anti-choline acetyltransferase (ChAT) antibody (1:1000; Millipore), a biotinylated donkey anti-goat IgG (1:1000; Jackson Immunoresearch Laboratories) and avidin/biotin complex (ABC) reagent (Vector Elite Kit: Vector Laboratories). ChAT-labeled cytoplasm and dendrites were revealed by a nickel-intensified diaminobenzidine (Ni-DAB) reaction that stained neurons black. Mounted sections were dehydrated and coverslipped with DePeX (Sigma-Aldrich).

Fluorescence immunohistochemistry was performed on free floating coronal sections to identify parvalbumin-positive neurons in the basal forebrain, and amyloid plaques and GFP-positive cells in the hippocampus and cortex. Briefly, sections were incubated overnight in mouse anti-parvalbumin antibody (1:200; Millipore), mouse anti-Aβ 6E10 (BioLegend; 1:1000) and rabbit anti-GFP (Abcam; 1:1000) followed by Alexa Fluor 488 or 647 (1:1000; Invitrogen) and DAPI (1:4000; Thermo Scientific) for 2 h. They were then washed and coverslipped with mounting medium for fluorescence (Dako).

Alternatively, hippocampal and cortical Aβ plaques were identified by Thioflavin-S staining. The sections were incubated in PBS-Triton X-100 with DAPI (1:4000; Thermo Scientific) and then washed in PBS-Triton X-100 before incubation in the dark for 5 min in twice-filtered 0.1% Thioflavin-S solution. The slides were then washed in 70% ethanol followed by distilled water to remove excess stain, after which they were allowed to dry before being coverslipped with fluorescence mounting medium (Dako).

Fluorescence and bright-field microscopy were performed using a fluorescence slide scanner (Axio, Zeiss) and a bright-field slide scanner (Axio, Zeiss), respectively.

### Quantification of Immunohistochemistry

The numbers of ChAT- and parvalbumin-positive neurons in the basal forebrain were counted for each animal, as per Boskovic et al. (2014). Briefly, anatomically matched sections (10 coronal sections per animal) containing the medial septum and diagonal bands of Broca were analyzed (beginning at 1.18 mm anterior to Bregma). Bilateral ChAT-and parvalbumin-labeled cells were counted manually within Imaris 7.2.3 software (Bitplane), and statistics were performed on the average of the total cells counted for each group using Graphpad Prism 6.

Quantification and measurements of amyloid plaques were obtained using a custom pipeline in CellProfiler, and the hippocampal area was calculated in Fiji (ImageJ). For each animal, every third hippocampal section (10 sections per animal) was counted, starting from the beginning of the hippocampus (1.06 mm posterior to Bregma).

### Measurement of Aβ and BDNF by ELISA

The levels of human Aβ_42_ (Invitrogen) and mouse BDNF (Biosensis) in supernatant prepared from hippocampal homogenates were measured using commercial ELISA kits.

Human Aβ_42_ levels were measured following extraction from hippocampal lysate. Briefly, hippocampal homogenate was homogenized in 5 M guanidine-HCl (diluted in 50 mM Tris, pH 8.0) as per the manufacturer’s instructions (Invitrogen). A bicinchoninic acid (BCA) assay (Thermo Scientific) to measure the total protein content was performed to ensure that the soluble lysate concentrations were within the assay range and could be standardized. The solid phase sandwich Aβ_42_ ELISA was performed according to the manufacturer’s instructions (Invitrogen) using a monoclonal antibody specific for the NH2-terminus of human Aβ and a rabbit antibody specific for the COOH-terminus of the 1–42 Aβ sequence, with the resultant measurements (pg) being normalized per mg of total soluble protein.

To measure BDNF levels, soluble proteins were extracted using an acid extraction protocol as per the manufacturer’s instructions. Briefly, hippocampi were suspended in 20 volume/weight extraction buffer (0.05 M sodium acetate, 1 M sodium chloride, 1% Triton-X100, Roche complete inhibitor cocktail tablet) and homogenized. A BCA assay (Thermo Scientific) was performed, followed by an ELISA for BDNF according to the manufacturer’s instructions (Biosensis). The resulting measurements (pg) were normalized per mg of total soluble protein.

### Sequential Extraction of Tau Protein

To measure the levels of tau and hyperphosphorylated tau epitopes, soluble (in reassembly buffer (RAB)) and insoluble (RIPA) fractions of hippocampal homogenate were sequentially extracted as previously described (Probst et al., 2000). Briefly, hippocampi were suspended in 10 volumes/weight ice-cold RAB buffer (0.01 M MES, 1 mM EGTA, 0.5 mM MgSO_4_, 0.75 M NaCl, 0.02 M NaF, 1 mM Na_3_VO_4_, 1 mM PMSF) containing Complete EDTA-free Protease Inhibitor Cocktail (Roche) and PhosSTOP Phosphatase Inhibitor Cocktail (Roche), and homogenized. Samples were centrifuged at 21,000 *g* for 90 min at 4°C and the supernatant was extracted as the RAB fraction. The remaining pellet was resuspended and homogenized in the same volume of ice-cold RIPA buffer (Cell Signaling) with 0.02 M NaF, 1 mM Na_3_VO_4_, 1 mM PMSF, Complete EDTA-free Protease Inhibitor Cocktail (Roche) and PhosSTOP Phosphatase Inhibitor Cocktail (Roche). Samples were centrifuged at 21,000 *g* for 90 min at 4°C and the supernatant was extracted as the RIPA fraction. A BCA assay (Thermo Scientific) was performed in order to measure the total protein content for each sample from each fraction to ensure that changes in tau were comparable across samples.

### Western Blotting

Total tau and hyperphosphorylated tau epitopes were quantified from the soluble and insoluble fractions of hippocampal and cortical homogenates by western blot analysis. Equal amounts of protein per mouse were separated on a NuPage Novex Bis-Tris Protein Gel (Life Technologies), and then transferred onto an Immobilon-FL transfer membrane (Millipore). The membranes were placed in blocking solution before incubation in the following antibodies: mouse AT8 (pSer202/pThr205; 1:1000; Thermo Scientific), mouse AT180 (pThr231; 1:1000; Thermo Scientific), mouse AT270 (pThr181; 1:1000; Thermo Scientific), rabbit pSer235 (1:1000; Thermo Scientific), rabbit pSer262 (1:1000; Thermo Scientific), rabbit pSer422 (1:1000; Thermo Scientific), mouse Tau5 (1:1000; Merck Millipore), and mouse and rabbit GAPDH (1:5000; Cell Signaling). The membranes were washed, then incubated with either anti-rabbit Alexa Fluor 680 (1:50,000; Invitrogen) or anti-mouse Alexa Fluor 800 (1:50,000; Invitrogen) antibodies. Protein bands were imaged using an Odyssey Imaging System (LI-COR Biosciences), and Image Studio software (LI-COR Biosciences) was used for quantification of western blots.

Levels of ChAT, and phosphorylated and total levels of the TrkB receptor were quantified from hippocampal homogenates by western blot analysis as described above. The following primary antibodies were used: goat anti-ChAT (1:1000; Invitrogen), rabbit anti-phosphoTrkB (phospho S478; 1:1000; Biosensis), goat anti-TrkB (1:1000; R&D Systems) and the loading control mouse anti-GAPDH (1:4000; Cell Signaling). Imaging and analysis were performed as described above.

### Y Maze

To measure short-term working memory, mice were tested in a two trial forced Y maze paradigm. The Y maze consisted of an elevated Y-shaped maze in clear plexi-glass with arms that were 40 cm long, 9 cm wide and 21 cm high. A removable barrier was used to shut off a maze arm of choice (randomized across genotype) during habituation. Extra-maze visual cues were placed around the room and the maze was wiped with ethanol after every trial to remove odor cues. The test was divided into two phases: a 5 min habituation in which mice could freely explore two arms of the maze, a 20 min interphase interval in the home cage, and a 5 min test phase in which all three arms were accessible.

In these tests, no group of mice showed a significant preference for the novel arm over the previously explored arms in terms of total time spent in the arm or their first entry choice (data not shown). A subgroup of 10 (five per condition) APP/PS1 transgenic mice were tested 1 month post-BFCN lesion in the Y maze (another group of 16 mice were tested in the Morris water maze, see below). As one mouse spent a considerable portion of the habituation trial immobile, its data were not included in the subsequent analysis. The APP/PS1 × BDNF^fl/wt^ mice were tested at 7 or 9 months of age, 2 months after AAV injection. Data were collected and analyzed using Ethovision XT (Noldus).

### Morris Water Maze

Spatial memory was assessed using the Morris water maze as previously described (Turnbull and Coulson, 2017). Briefly, a subgroup of APP/PS1 mice were placed in a circular pool filled with opaque water with four virtual quadrants indicated by cues mounted on the surrounding walls. The mice underwent a 5 day acquisition phase in which they were released from three different start positions per day, with 20 min intervals between each training run. On the 6th day, the mice were subjected to a probe trial (in which the platform was removed) to test spatial memory retention. To investigate BFCN-mediated spatial learning, the platform was moved to a new location and the mouse’s ability to “update” its memory of the platform location was assessed over the 7th and 8th days (reversal task). A probe trial was again performed on the 9th day.

Latency was measured as the time from when the mouse was placed in the water until it had remained in the arena for a maximum of 60 s or until it had remained on the platform for a total of 10 s. Data were collected and analyzed using Ethovision XT (Noldus).

### Statistics

All data are expressed as mean ± SEM. Statistical tests, including appropriate *post hoc* analysis, were performed for each result and are reported in the figure legends. The significance threshold was set at *p* < 0.05 and analyses were performed using Graphpad Prism 6.

### Data Availability

The datasets generated during and/or analyzed during the current study are available from the corresponding author on reasonable request.

## Results

### p75-Saporin Toxin Injection into the Basal Forebrain Selectively Lesions Cholinergic Neurons

**Figure 1:**
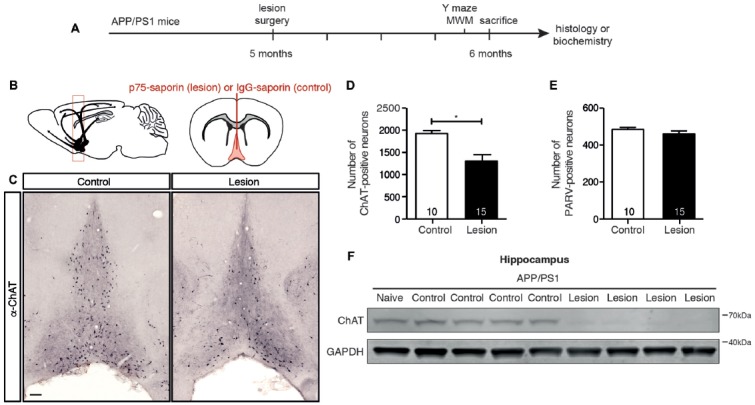
p75-saporin toxin injection into the basal forebrain selectively lesions cholinergic neurons.** (A)** Representative schematic of the paradigm indicating the age and time of surgery and experimental manipulations. **(B)** Diagram of a murine brain in the sagittal plane (left), with a red box indicating the location of the basal forebrain, and the coronal plane (right), indicating the location of the saporin injection site in the medial septum region of the basal forebrain (in red). **(C)** Representative photomicrographs of coronal brain sections at the level of the basal forebrain, immunostained for a cholinergic neuron marker (anti-ChAT) 1 month after microinjection of IgG-saporin (control) or p75-saporin (lesion) into the medial septum. **(D)** Quantification of ChAT- and **(E)** parvalbumin (PARV)-positive neurons in the basal forebrain of basal forebrain cholinergic neuron (BFCN)-lesioned and control transgenic mice. The number of animals per condition are indicated within the bars of the graphs. *p < 0.05, unpaired *t*-test. **(F)** Representative western blots of ChAT protein levels in the hippocampus of naïve, unlesioned (control) and BFCN-lesioned amyloid precursor protein (APP)/PS1 transgenic mice. Data are presented as mean ± SEM.

In order to selectively ablate the cholinergic septo-hippocampal pathway, a single injection of p75-saporin, or its non-specific IgG-saporin control, was stereotaxically injected into the medial septum of 5 month old APP/PS1 transgenic mice (Figures 1A,B). As p75^NTR^ expression is restricted to cholinergic neurons in the basal forebrain, endocytosis of the toxin induces the selective death of cholinergic neurons. One month post-injection, the basal forebrain was assessed for cholinergic neuron loss by counting the number of ChAT-positive neurons in histologic sections (Figure 1C). p75-saporin injection resulted in a 40% loss of BFCNs (Figure 1D). The selectivity of the toxin was verified by counting the number of parvalbumin-positive neurons. As expected, no change in the number of these neurons, which do not express p75^NTR^, was observed (Figure 1E). The 40% loss of cholinergic neurons caused by the lesion was sufficient to reduce cholinergic innervation to the hippocampus, as western blot analysis of hippocampal homogenates from lesioned animals revealed a significant decrease in the level of ChAT protein (Figure 1F).

### Memory Retention in a Morris Water Maze Task Is Impaired Following Basal Forebrain Cholinergic Neuron Lesion in APP/PS1 Mice

**Figure 2:**
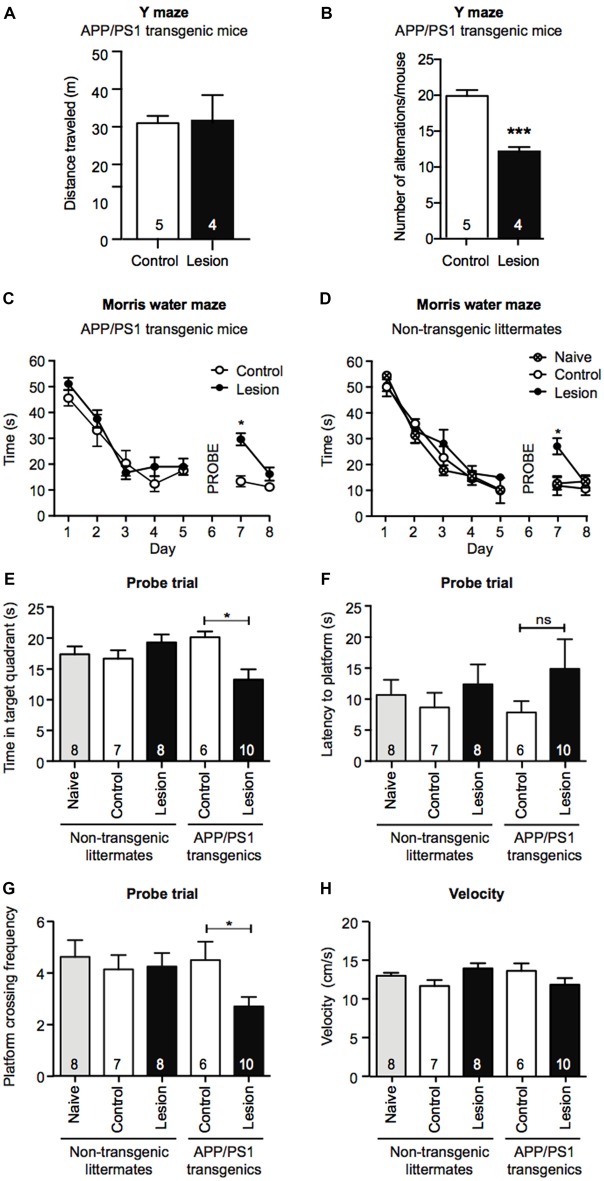
Memory retention is impaired in a Morris water maze task following BFCN lesion in APP/PS1 mice. Graphs of total distance traveled **(A)** and number of maze arm alternations **(B)** of basal forebrain-lesioned (p75-saporin) APP/PS1 mice and their non-lesioned (IgG-saporin) transgenic controls (mean ± SEM, ***p < 0.001, unpaired *t*-test). Graph of the escape latency (time to find the hidden platform) averaged per day in an 8 day Morris water maze task of spatial learning of **(C)** basal forebrain-lesioned (p75-saporin) APP/PS1 mice and their non-lesioned (IgG-saporin) transgenic controls, and **(D)** basal forebrain-lesioned (p75-saporin) non-lesioned (IgG-saporin) and naïve non-transgenic littermates of the same age. A probe trial in which the platform was removed was performed on day 6. The platform location was then changed for testing on days 7 and 8. Two-way analysis of variance (ANOVA) with Tukey’s *post hoc* test, *p < 0.05. **(E)** Quantification of time spent in the target quadrant, **(F)** latency to the platform, and **(G)** platform crossing frequency during the probe trial on day 6, between basal forebrain-lesioned and non-lesioned APP/PS1 transgenic mice and basal forebrain-lesioned, non-lesioned, and naïve non-transgenic littermate controls. **(H)** Quantification of velocity during the acquisition stage. Data are presented as mean ± SEM with sample size indicated in the bars. One-way ANOVA with Tukey’s *post hoc*, *p < 0.05, ns, not significant.

To determine if BFCN lesions in APP/PS1 transgenic mice accelerated cognitive decline, we first tested 6 month old mice for working memory in the Y maze 1 month post-injection of IgG- or p75-saporin into the basal forebrain. Although there was no significant difference in the distance each group of mice traveled (Figure 2A), BFCN lesioned mice displayed a significant reduction in spontaneous entries into alternate arms of the maze compared to non-lesioned mice (Figure 2B), indicating a degree of hippocampal dysfunction.

Although APP/PS1 transgenic mice display obvious cognitive impairments by 12 months of age, at 6 months of age their performance is comparable to that of wild-type mice in the hippocampal-dependent spatial learning task, the Morris water maze (Lalonde et al., 2005; Volianskis et al., 2010). To test for changes in long-term memory in the Morris water maze paradigm, we compared the task performance of IgG- and p75-saporin-injected APP/PS1 transgenic mice with age-, gender-, and treatment-matched non-transgenic littermates, together with naïve (no stereotaxic surgery) non-transgenic mice. During the 5 day acquisition phase of the task, all experimental groups demonstrated learning as measured by a significant decrease in the time taken to reach the hidden escape platform. This indicates that BFCN lesioning in both non-transgenic and APP/PS1 transgenic mice does not impact learning acquisition at this age (Figures 2C,D). However, in the probe trial, BFCN-lesioned APP/PS1 mice spent less time in the target quadrant, and made fewer crosses over the platform location than non-lesioned APP/PS1 mice or their non-transgenic littermates, regardless of BFCN lesion status (Figures 2E–G).

On days 7 and 8, all experimental groups underwent a reversal test where the platform location was changed. Non-transgenic littermates and APP/PS1 transgenic mice that had BFCN lesions took longer to locate the platform than unlesioned and naïve mice (Figures 2C,D). This is consistent with previous reports in which “updating” of memory is linked to basal forebrain function (Tait and Brown, 2008; Al-Onaizi et al., 2017). Thus, a deficit in reversal task learning serves as an internal control for an effective BFCN lesion. All experimental mice traveled equivalent distances in the maze and there were no obvious differences in velocity (Figure 2H), ruling out altered motor function as an explanation for the above results. Taken together, these results indicate that the combination of the APP/PS1 genotype and the BFCN lesion resulted in an impairment of the cognitive processes required for optimal memory performance in the Morris water maze and Y maze tasks.

### Total Aβ_42_ Levels in the Hippocampus Increase Following Basal Forebrain Cholinergic Neuron Lesion

**Figure 3:**
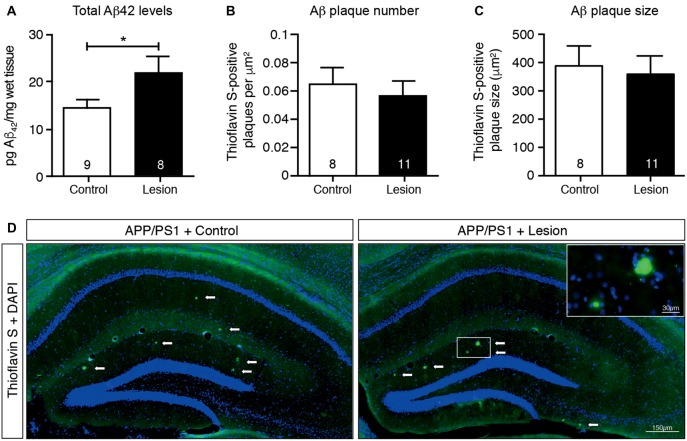
Total Aβ_42_ levels increase in the hippocampus following BFCN lesion.** (A)** Quantification of total Aβ_42_ levels by ELISA from hippocampal lysates of control and basal forebrain-lesioned APP/PS1 mice. Quantification of **(B)** Aβ plaque number with Thioflavin S staining per μm^2^ and **(C)** plaque size across the complete hippocampus of basal forebrain-lesioned and control mice. Loss of BFCNs had no effect on plaque number or size in the hippocampus but resulted in an increase in total Aβ_42_ levels in the hippocampus. Data are presented as mean ± SEM, with sample size indicated in the bars. Unpaired *t*-test, *p < 0.05. **(D)** Representative fluorescence images of amyloid plaques (visualized with Thioflavin S staining) in the hippocampus of APP/PS1 transgenic mice with IgG-saporin injection (control) or p75-saporin injection (lesion) into the basal forebrain. Plaques are indicated by white arrows and a magnified view of a plaque is visible in the top right corner.

The APP/PS1 transgenic mouse line carries human APP and PS1 (presenilin 1) transgenes that harbor mutations associated with early onset AD, as a result of which Aβ (particularly the aggregation-prone longer form, Aβ_42_) is overproduced and accumulates as plaques in the brains of these animals (Jankowsky et al., 2004). To determine if cholinergic denervation exacerbated this accumulation of Aβ, we lesioned BFCNs in APP/PS1 transgenic mice and measured the Aβ level in hippocampal homogenates by ELISAs, as well as determining the size and number of Aβ plaques in histological sections. Our results revealed an increase in total Aβ_42_ levels in the hippocampal lysates following BFCN lesion that was not observed in IgG-saporin-injected mice (Figure 3A). However, this increase in total Aβ_42_ was not reflected in hippocampal amyloid plaque number or size (Figures 3B–D). Indeed there were only rare amyloid deposits in the hippocampus of these 6 month old mice (Figure 3D). The size and number of cortical plaques was also determined, but again, no changes were observed following BFCN lesion (data not shown). These results suggest that cholinergic degeneration exacerbates the production of soluble Aβ_42_, which has not yet accumulated and been deposited into plaques.

**Figure 4:**
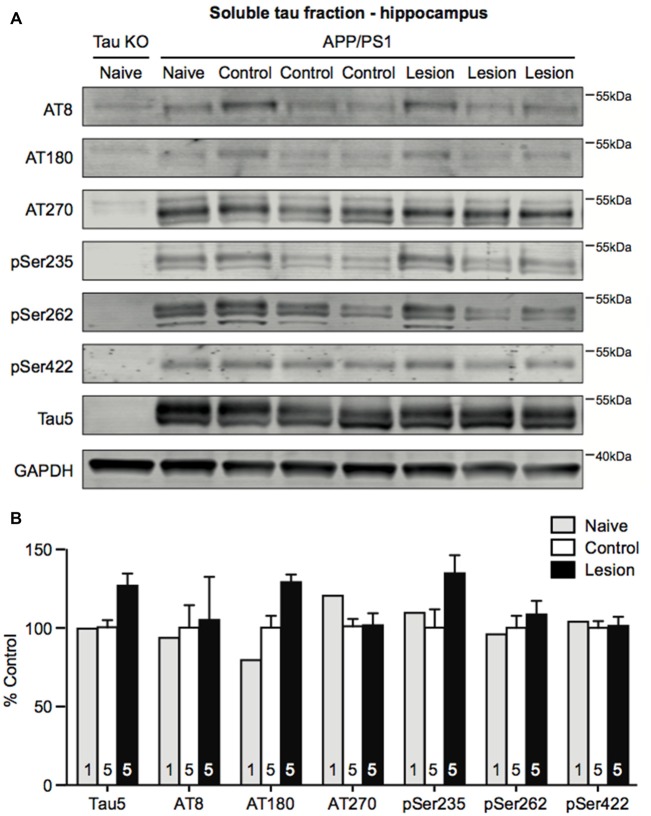
Tau phosphorylation does not change in the hippocampus following BFCN lesion.** (A)** Representative western blots of the soluble tau fraction of hippocampal homogenates from naïve tau knockout mice, and naïve, control (IgG-saporin) and basal forebrain-lesioned (p75-saporin) APP/PS1 mice probed for the pan-tau marker Tau5 and the tau hyperphosphorylation epitopes AT8, AT180, AT270, pSer235, pSer262 and pSer422. Each tau band is compared to its respective GAPDH loading control band. **(B)** Quantification of tau protein bands from the soluble tau fraction. No significant differences (two-way ANOVA) in hyperphosphorylated tau epitopes were observed between naïve, control and basal forebrain-lesioned APP/PS1 mice. Bands were normalized to the GAPDH loading control levels and the levels are plotted relative to those of control APP/PS1 mice. Data are presented as the mean ± SEM, with the sample size indicated in the bars.

Ramos-Rodriguez et al. (2013) have previously reported an increase in a single hyperphosphorylated tau epitope in the cortex following BFCN lesion of APP/PS1 transgenic mice. To assess whether tau pathology was also exacerbated in APP/PS1 transgenic mice following loss of BFCNs, we performed sequential extraction of tau protein and measured the levels by western blot analysis of hippocampal homogenates. The levels of total tau and a range of tau phosphorylation epitopes that are considered to be markers of pathological hyperphosphorylation were assessed. However, no changes in the levels of pan-tau or a variety of phospho-tau epitopes in the soluble (Figures 4A,B) and insoluble (data not shown) fractions were observed following BFCN lesion of the transgenic mice.

### A Reduction in BDNF Protein and TrkB Receptor Signaling Are Observed Following Basal Forebrain Cholinergic Neuron Lesion

**Figure 5:**
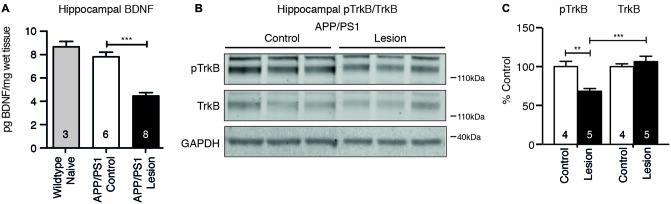
Brain-derived neurotrophic factor (BDNF) protein and TrkB receptor signaling are reduced following BFCN lesion.** (A)** Quantification of BDNF protein levels by ELISA from hippocampal lysates of naïve wild-type mice and control and basal forebrain-lesioned APP/PS1 mice. Loss of BFCNs resulted in a decrease in the BDNF protein level. **(B)** Representative western blots of hippocampal homogenates from control (IgG-saporin) and basal forebrain-lesioned (p75-saporin) APP/PS1 mice probed for phosphorylated TrkB (pTrkB) and total TrkB. Each band is compared to its respective GAPDH loading control band. **(C)** Quantification of pTrkB and TrkB bands expressed as a percentage of the levels in control mice. Loss of the cholinergic projection to the hippocampus resulted in a reduction in phosphorylated/active TrkB receptor. Data are presented as mean ± SEM, with the sample size indicated in the bars. One-way ANOVA, **p < 0.01, ***p < 0.001.

To test whether reduced cholinergic innervation to the hippocampus following BFCN lesion affected neurotrophic signaling, the level of BDNF protein was measured by ELISA, while TrkB receptor activation was measured by western blot analysis. BFCN lesion in APP/PS1 transgenic mice resulted in a reduction in the level of BDNF protein in the hippocampus (Figure 5A). This decrease was confirmed by a reduction in the level of phosphorylated TrkB, reflecting reduced activation of the receptor (Figures 5B,C).

### A Reduction in the Anterior Hippocampal BDNF Level Does Not Affect Working Memory

**Figure 6:**
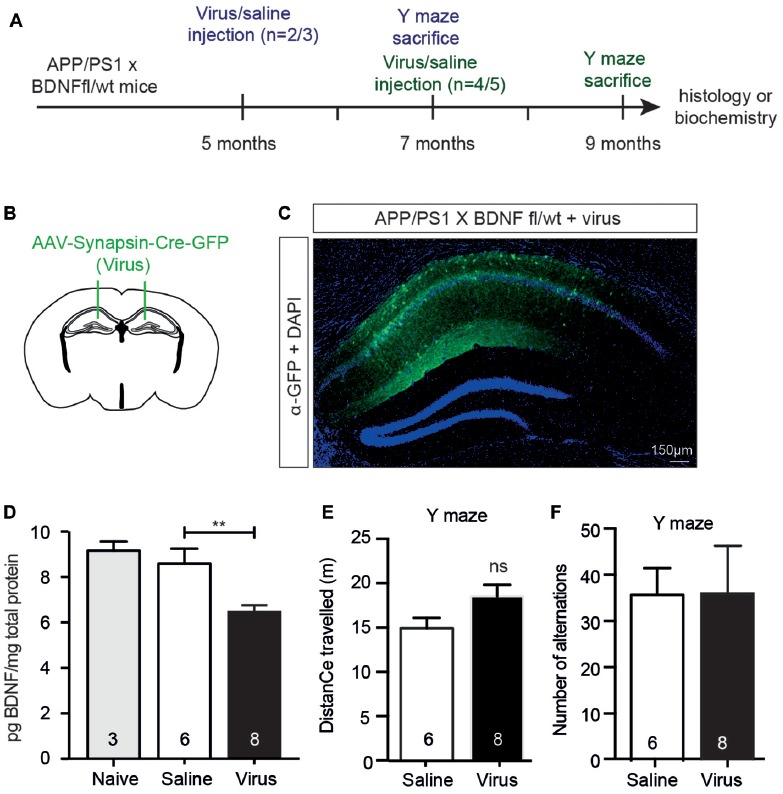
A selective reduction in the hippocampal BDNF level does not alter working memory.** (A)** Representative schematic of the paradigm indicating the age and time of surgery and the experimental manipulations. **(B)** Diagram of a murine brain in the coronal plane with green lines indicating the location of the stereotaxic injection of virus (AAV-Synapsin-Cre-GFP). **(C)** Representative fluorescence image of a hippocampal cross-section demonstrating the spatial distribution of the virus carrying a GFP tag (in green) after stereotaxic injection into an APP/PS1 × BDNF^fl/wt^ mouse. **(D)** Quantification of the BDNF protein levels (pg/mg total protein) by ELISA from anterior hippocampal lysates of naïve (gray bars), saline-injected and virus-injected APP/PS1 × BDNF^fl/wt^ mice. Stereotaxic injection of AAV-Synapsin-Cre-GFP into the hippocampus of mice carrying a floxed BDNF allele resulted in a decrease in BDNF protein. One-way ANOVA, **p < 0.01. Graphs of total distance traveled **(E)** and the number of maze arm alternations **(F)** of APP/PS1 × BDNF^fl/wt^ mice injected with a saline control or AAV Synapsin-Cre virus (unpaired *t*-test), ns, not significant.

To test whether the effects seen following BFCN lesioning were due to the reduction in hippocampal BDNF, we reduced BDNF expression to a level equivalent to that observed in BFCN-lesioned mice by injecting a neuron-specific AAV-Synapsin-Cre-GFP bilaterally into the anterior hippocampus of 5 or 7 month old APP/PS1 × BDNF^fl/wt^ mice (Figures 6A,B). Six weeks after injection of the virus, we assessed its spread by staining histological sections with an anti-GFP antibody, revealing labeling throughout the CA1/CA2 subregions of the anterior hippocampus, but not in the dentate gyrus (Figure 6C). The BDNF protein levels in homogenates of the anterior hippocampus of virus-injected animals were measured by ELISA, revealing a significant decrease compared to saline-injected controls and naïve animals (Figure 6D). To assess whether this had an effect on memory, mice were assessed in the Y maze 2 months after their surgery. No difference in the total distance traveled or alternation was observed between saline- and virus-injected APP/PS1 × BDNF^fl/wt^ mice (Figures 6E,F).

### Reduction of Anterior Hippocampal BDNF Levels Do Not Alter Aβ Levels or Affect Amyloid Pathology

**Figure 7:**
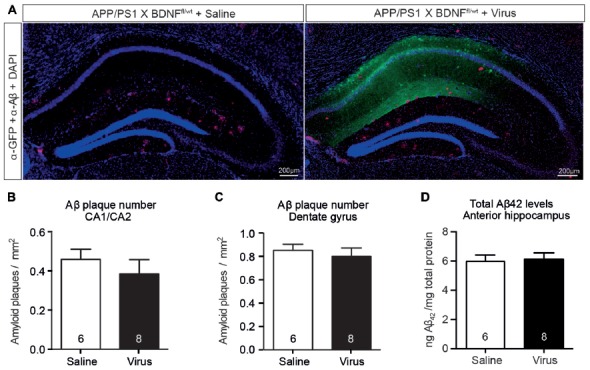
A selective reduction in the hippocampal BDNF level does not alter the level of Aβ or affect amyloid pathology.** (A)** Representative fluorescence images of the hippocampus of APP/PS1 × BDNF^fl/wt^ mice injected with a saline control or virus. Amyloid plaques were labeled with an Aβ antibody (red, arrowed), and cells infected with the virus were labeled with a GFP antibody (green). Quantification of the number of amyloid plaques in **(B)** the CA1/CA2 and **(C)** the dentate gyrus subregions of the hippocampus of APP/PS1 × BDNF^fl/wt^ mice injected with a saline control or virus. **(D)** Quantification of total Aβ_42_ levels by ELISA from anterior hippocampal lysates of saline- and virus-injected APP/PS1 × BDNF^fl/wt^ mice. A reduction in the BDNF protein level in the hippocampus did not affect the number of amyloid plaques, or the level of soluble Aβ. Data are presented as mean ± SEM, with the sample size indicated in the bars.

We next determined if the reduced hippocampal BDNF protein levels (in the absence of coincident cholinergic dysfunction) induced an increase in hippocampal Aβ_42_ levels in APP/PS1 × BDNF^fl/wt^ mice injected with AAV-Synapsin-Cre-GFP. There was no change in plaque number or size in either the area of the hippocampus infected with virus, as indicated by immunostaining for GFP, or in non-infected areas (Figures 7A–C). The total Aβ_42_ levels were also unchanged in lysates of the anterior hippocampus between mice injected with AAV-Synapsin-Cre-GFP or saline (Figure 7D). These results suggest that, although BDNF signaling is reduced following BFCN lesion in APP/PS1 mice, reduced BDNF levels alone do not exacerbate the accumulation of Aβ.

## Discussion

Degeneration of BFCNs is an early and core feature of sporadic AD that underpins aspects of cognitive deterioration and contributes to disease progression (Grothe et al., 2010; Kerbler et al., 2015; Schmitz and Nathan Spreng, 2016). In genetically susceptible mouse models, inducing cholinergic degeneration can exacerbate the production and/or accumulation of Aβ, and cause cognitive impairment (Gil-Bea et al., 2012; Laursen et al., 2013; Ramos-Rodriguez et al., 2013; Hartig et al., 2014), as well as resulting in reduced levels of BDNF, another feature of AD (Egan et al., 2003; Tapia-Arancibia et al., 2008; Fahnestock, 2011). However, the etiological relationship between lowered BDNF levels and Aβ accumulation are unclear. Here we show that although BFCN lesions in adult mice induce reduced neurotrophin signaling, this loss of neuronal BDNF is unlikely to be directly responsible for the coincident increase in Aβ accumulation.

We report that loss of cholinergic neurons in young presymptomatic Aβ-overproducing mice triggers an increase in the level of soluble Aβ_42_ in the hippocampus, a post-synaptic target of BFCNs, causing cognitive dysfunction in two hippocampal-dependent spatial navigation tasks. This finding is consistent with previous reports which demonstrated exacerbation of Aβ pathology and cognitive impairment in AD model mice (Gil-Bea et al., 2012; Laursen et al., 2013; Ramos-Rodriguez et al., 2013; Hartig et al., 2014). However, the memory deficits we observed in the probe trial of the Morris water maze 1 month after BFCN lesioning of 5 month old APP/PS1 mice occurred at an earlier age than previously reported, with Ramos-Rodriguez et al. (2013) describing deficits in 7 month old mice that had harbored BFCN lesions for 4 months. In both studies, BFCN lesions in wild-type mice did not cause equivalent memory deficits, indicating that cholinergic denervation (including BDNF down-regulation) alone is insufficient to impair memory retention. The most likely explanation for the observed memory deficits in the APP/PS1 BFCN-lesioned mice is the increase in soluble Aβ levels observed in our study (this was not measured in the Ramos-Rodriguez et al.’s (2013) study). This is also a possible explanation for the Y maze alternation deficits which were observed in lesioned but not APP/PS1 × BDNF ^fl/wt^ mice. However, similar phenotypes have been reported not only for AD model mice but in situations of cholinergic neurotransmission impairment and reduced BDNF levels (Kokkinidis and Anisman, 1976; Tempier et al., 2013; Chen et al., 2016). As the reduced BDNF may have contributed to the Aβ-induced cognitive impairment in the Morris water maze (Rantamäki et al., 2013), we asked whether the decrease in BDNF was required for the rise in Aβ levels.

Dysfunctional neurotrophic signaling is a feature of AD and a risk factor for cognitive decline (Peng et al., 2005; Fahnestock, 2011). In particular, the BDNF polymorphism resulting in a Val66Met substitution, which reduces the activity-dependent neuronal secretion of BDNF (Egan et al., 2003), accounts for up to 30% of the variability in cognitive decline in AD patients (Lim et al., 2013, 2015). In our experiments, the levels of hippocampal BDNF protein and TrkB receptor signaling were reduced following BFCN lesion, consistent with previous studies showing that a complete lesion of BFCNs in rats results in reduced BDNF mRNA in the hippocampus (Kokaia et al., 1996; Ferencz et al., 1997) and that cholinergic basal forebrain innervation of the hippocampus is involved in the regulation of BDNF protein and mRNA expression (Lapchak et al., 1993; Gil-Bea et al., 2011). However, despite achieving a significant decrease in hippocampal BDNF level in APP/PS1 × BDNF ^fl/wt^ mice injected with a synapsin Cre recombinase-carrying AAV, equivalent to that of a BDNF heterozygous animal, we did not observe an exacerbation in Aβ pathology or an increase in the level of Aβ.

Although in our study only neuronal BDNF was reduced, previous *in vivo* studies have reported that heterozygous BDNF;APP/PS1 mice display no alteration in amyloid plaque formation at 9 months of age (Rantamäki et al., 2013). Similarly, the levels of Aβ and tau pathology in both heterozygous BDNF;3XTg-AD mice and TrkB;3XTg-AD mice are reported to be comparable to those of transgenic controls (Castello et al., 2012). Why might we expect a different result in our experiments when BDNF is lost only from neurons? Loss of the BDNF gene from conception could lead to compensation; despite the 43% reduction in cortical BDNF protein reported by Castello et al. (2012) and Rantamäki et al. (2013), the authors also described an age-dependent increase in mature BDNF protein, with BDNF mRNA and phospho-TrkB levels being unaltered in the cortical tissue of the APP/PS1 mice. Acute loss of BDNF, such as occurs following BFCN lesion or damage, may therefore trigger different effects. *In vitro*, Matrone et al. (2008) demonstrated that complete withdrawal of BDNF from hippocampal neurons using a blocking antibody resulted in increased Aβ production. Therefore the acute change in BDNF levels and TrkB signaling in our study, equivalent to the BDNF loss that occurs in human AD patients (Tapia-Arancibia et al., 2008), could have induced increased Aβ production; however, we did not observe this. As there is now a considerable body of research, including work in humans (Lim et al., 2013, 2015), which indicates that neither a chronic nor an acute reduction of neuronal BDNF is sufficient to cause Aβ accumulation *in vivo*, it is reasonable to conclude that reduced BDNF levels do not directly cause AD.

What then is responsible for the increased Aβ levels that we and others have reported? Recent reports indicate that cholinergic deficiency, induced by knockout of the cholinergic vesicle transporter and mediated by reduced M1 muscarinic receptor signaling, could result in changes in BFCN-derived microRNAs and the splicing of the β-site APP cleaving enzyme 1 (BACE1) mRNA and BACE1 overexpression (Fisher, 2012; Kolisnyk et al., 2016, 2017). In turn, increased BACE activity would favor the amyloidogenic cleavage pathway, raising the level of soluble Aβ_42_. Furthermore, Aβ could be released by degenerating BFCNs, and neurotrophin signaling within BFCNs could control Aβ production. Therefore, there are a number of possible mechanisms by which cholinergic degeneration could lead directly to Aβ generation. We surmise that increased Aβ in combination with reduced BDNF levels results in cognitive dysfunction. In support of this idea, BDNF is known to be beneficial for synaptic and cognitive function, including in the presence of Aβ pathology. For example, Blurton-Jones et al. (2009) demonstrated that transplanted neural stem cell-derived BDNF increased the density of synapses in the hippocampus of 3XTg-AD mice and reversed cognitive deficits without alterations in Aβ or tau pathology. Therefore, although dysfunctional neurotrophin signaling does not appear to directly affect the level of Aβ *in vivo*, the therapeutic potential of enhancing neurotrophic signaling remains a viable option (Nagahara and Tuszynski, 2011).

## Author Contributions

All authors designed and analyzed experiments, MTT and ZB performed experiments and MTT and EJC wrote the manuscript.

## Conflict of Interest Statement

The authors declare that the research was conducted in the absence of any commercial or financial relationships that could be construed as a potential conflict of interest.

## References

[B1] Al-Onaizi M. A. Parfitt G. M. Kolisnyk B. Law C. S. Guzman M. S. Barros D. M.. (2017). Regulation of cognitive processing by hippocampal cholinergic tone. Cereb. Cortex 27, 1615–1628. 10.1093/cercor/bhv349 26803167

[B2] Arancibia S. Silhol M. Moulière F. Meffre J. Höllinger I. Maurice T.. (2008). Protective effect of BDNF against β-amyloid induced neurotoxicity *in vitro* and *in vivo* in rats. Neurobiol. Dis. 31, 316–326. 10.1016/j.nbd.2008.05.012 18585459

[B3] Blurton-Jones M. Kitazawa M. Martinez-Coria H. Castello N. A. Müller F. J. Loring J. F.. (2009). Neural stem cells improve cognition via BDNF in a transgenic model of Alzheimer disease. Proc. Natl. Acad. Sci. U S A 106, 13594–13599. 10.1073/pnas.0901402106 19633196 PMC2715325

[B4] Boskovic Z. Alfonsi F. Rumballe B. A. Fonseka S. Windels F. Coulson E. J. (2014). The role of p75NTR in cholinergic basal forebrain structure and function. J. Neurosci. 34, 13033–13038. 10.1523/JNEUROSCI.2364-14.2014 25253850 PMC6608337

[B5] Burgess B. L. Mcisaac S. A. Naus K. E. Chan J. Y. Tansley G. H. Yang J.. (2006). Elevated plasma triglyceride levels precede amyloid deposition in Alzheimer’s disease mouse models with abundant Aβ in plasma. Neurobiol. Dis. 24, 114–127. 10.1016/j.nbd.2006.06.007 16899370

[B6] Castello N. A. Green K. N. LaFerla F. M. (2012). Genetic knockdown of brain-derived neurotrophic factor in 3xTg-AD mice does not alter Aβ or tau pathology. J. Neuropathol. Exp. Neurol. 7:e39566. 10.1371/journal.pone.0039566 22870188 PMC3411687

[B7] Chen Z. Huang C. Ding W. (2016). Z-guggulsterone improves the scopolamine-induced memory impairments through enhancement of the BDNF signal in C57BL/6J mice. Neurochem. Res. 41, 3322–3332. 10.1007/s11064-016-2064-0 27677871

[B8] Contestabile A. (2011). The history of the cholinergic hypothesis. Behav. Brain Res. 221, 334–340. 10.1016/j.bbr.2009.12.044 20060018

[B9] Egan M. F. Kojima M. Callicott J. H. Goldberg T. E. Kolachana B. S. Bertolino A.. (2003). The BDNF val66met polymorphism affects activity-dependent secretion of BDNF and human memory and hippocampal function. Cell 112, 257–269. 10.1016/s0092-8674(03)00035-7 12553913

[B10] Elliott E. Atlas R. Lange A. Ginzburg I. (2005). Brain-derived neurotrophic factor induces a rapid dephosphorylation of tau protein through a PI-3 Kinase signalling mechanism. Eur. J. Neurosci. 22, 1081–1089. 10.1111/j.1460-9568.2005.04290.x 16176349

[B11] Fahnestock M. (2011). Brain-derived neurotrophic factor: the link between amyloid-β and memory loss. Future Neurol. 6, 627–639. 10.2217/fnl.11.44

[B12] Ferencz I. Kokaia M. Keep M. Elmér E. Metsis M. Kokaia Z.. (1997). Effects of cholinergic denervation on seizure development and neurotrophin messenger RNA regulation in rapid hippocampal kindling. Neuroscience 80, 389–399. 10.1016/s0306-4522(97)00006-7 9284342

[B13] Fisher A. (2012). Cholinergic modulation of amyloid precursor protein processing with emphasis on M1 muscarinic receptor: perspectives and challenges in treatment of Alzheimer’s disease. J. Neurochem. 120, 22–33. 10.1111/j.1471-4159.2011.07507.x 22122190

[B14] Gil-Bea F. J. Gerenu G. Aisa B. Kirazov L. P. Schliebs R. Ramírez M. J. (2012). Cholinergic denervation exacerbates amyloid pathology and induces hippocampal atrophy in Tg2576 mice. Neurobiol. Dis. 48, 439–446. 10.1016/j.nbd.2012.06.020 22759926

[B15] Gil-Bea F. J. Solas M. Mateos L. Winblad B. Ramírez M. J. Cedazo-Mínguez A. (2011). Cholinergic hypofunction impairs memory acquisition possibly through hippocampal Arc and BDNF downregulation. Hippocampus 21, 999–1009. 10.1002/hipo.20812 20865740

[B16] Grothe M. Heinsen H. Teipel S. (2013). Longitudinal measures of cholinergic forebrain atrophy in the transition from healthy aging to Alzheimer’s disease. Neurobiol. Aging 34, 1210–1220. 10.1016/j.neurobiolaging.2012.10.018 23158764 PMC4058576

[B17] Grothe M. Zaborszky L. Atienza M. Gil-Neciga E. Rodriguez-Romero R. Teipel S. J.. (2010). Reduction of basal forebrain cholinergic system parallels cognitive impairment in patients at high risk of developing Alzheimer’s disease. Cereb. Cortex 20, 1685–1695. 10.1093/cercor/bhp232 19889714 PMC2912653

[B18] Hall A. M. Moore R. Y. Lopez O. L. Kuller L. Becker J. T. (2008). Basal forebrain atrophy is a presymptomatic marker for Alzheimer’s disease. Alzheimers Dement. 4, 271–279. 10.1016/j.jalz.2008.04.005 18631978 PMC2517158

[B19] Hartig W. Saul A. Kacza J. Grosche J. Goldhammer S. Michalski D.. (2014). Immunolesion-induced loss of cholinergic projection neurones promotes β-amyloidosis and tau hyperphosphorylation in the hippocampus of triple-transgenic mice. Neuropathol. Appl. Neurobiol. 40, 106–120. 10.1111/nan.12050 23566195

[B20] Holsinger R. M. Schnarr J. Henry P. Castelo V. T. Fahnestock M. (2000). Quantitation of BDNF mRNA in human parietal cortex by competitive reverse transcription-polymerase chain reaction: decreased levels in Alzheimer’s disease. Mol. Brain Res. 76, 347–354. 10.1016/s0169-328x(00)00023-1 10762711

[B21] Jankowsky J. L. Fadale D. J. Anderson J. Xu G. M. Gonzales V. Jenkins N. A.. (2004). Mutant presenilins specifically elevate the levels of the 42 residue β-amyloid peptide *in vivo*: evidence for augmentation of a 42-specific γ secretase. Hum. Mol. Genet. 13, 159–170. 10.1093/hmg/ddh019 14645205

[B22] Kerbler G. M. Fripp J. Rowe C. C. Villemagne V. L. Salvado O. Rose S.. (2015). Basal forebrain atrophy correlates with amyloid β burden in Alzheimer’s disease. Neuroimage Clin. 7, 105–113. 10.1016/j.nicl.2014.11.015 25610772 PMC4299972

[B23] Kokaia M. Ferencz I. Leanza G. Elmér E. Metsis M. Kokaia Z.. (1996). Immunolesioning of basal forebrain cholinergic neurons facilitates hippocampal kindling and perturbs neurotrophin messenger RNA regulation. Neuroscience 70, 313–327. 10.1016/0306-4522(95)00384-3 8848142

[B24] Kokkinidis L. Anisman H. (1976). Interaction between cholinergic and catecholaminergic agents in a spontaneous alternation task. Psychopharmacology 48, 261–270. 10.1007/bf00496859 823581

[B25] Kolisnyk B. Al-Onaizi M. Soreq L. Barbash S. Bekenstein U. Haberman N.. (2017). Cholinergic surveillance over hippocampal RNA metabolism and Alzheimer’s-like pathology. Cereb. Cortex 27, 3553–3567. 10.1093/cercor/bhw177 27312991

[B26] Kolisnyk B. Al-Onaizi M. A. Xu J. Parfitt G. M. Ostapchenko V. G. Hanin G.. (2016). Cholinergic regulation of hnRNPA2/B1 translation by M1 muscarinic receptors. J. Neurosci. 36, 6287–6296. 10.1523/JNEUROSCI.4614-15.2016 27277805 PMC6604881

[B27] Lalonde R. Kim H. D. Maxwell J. A. Fukuchi K. (2005). Exploratory activity and spatial learning in 12-month-old *APP*_(695)_*SWE*/co+*PS1*/ΔE9 mice with amyloid plaques. Neurosci. Lett. 390, 87–92. 10.1016/j.neulet.2005.08.028 16169151

[B28] Lapchak P. A. Araujo D. M. Hefti F. (1993). Cholinergic regulation of hippocampal brain-derived neurotrophic factor mRNA expression: evidence from lesion and chronic cholinergic drug treatment studies. Neuroscience 52, 575–585. 10.1016/0306-4522(93)90407-7 8450959

[B29] Laske C. Stellos K. Hoffmann N. Stransky E. Straten G. Eschweiler G. W.. (2011). Higher BDNF serum levels predict slower cognitive decline in Alzheimer’s disease patients. Int. J. Neuropsychopharmacol. 14, 399–404. 10.1017/S1461145710001008 20860877

[B30] Laske C. Stransky E. Leyhe T. Eschweiler G. W. Wittorf A. Richartz E.. (2006). Stage-dependent BDNF serum concentrations in Alzheimer’s disease. J. Neural Transm. 113, 1217–1224. 10.1007/s00702-005-0397-y 16362629

[B31] Laursen B. Mørk A. Plath N. Kristiansen U. Bastlund J. F. (2013). Cholinergic degeneration is associated with increased plaque deposition and cognitive impairment in APPswe/PS1dE9 mice. Behav. Brain Res. 240, 146–152. 10.1016/j.bbr.2012.11.012 23178660

[B32] Lim Y. Y. Villemagne V. L. Laws S. M. Ames D. Pietrzak R. H. Ellis K. A.. (2013). BDNF Val66Met, Aβ amyloid, and cognitive decline in preclinical Alzheimer’s disease. Neurobiol. Aging 34, 2457–2464. 10.1016/j.neurobiolaging.2013.05.006 23769397

[B33] Lim Y. Y. Villemagne V. L. Laws S. M. Pietrzak R. H. Snyder P. J. Ames D.. (2015). APOE and BDNF polymorphisms moderate amyloid β-related cognitive decline in preclinical Alzheimer’s disease. Mol. Psychiatry 20, 1322–1328. 10.1038/mp.2014.123 25288138 PMC4759101

[B34] Matrone C. Ciotti M. T. Mercanti D. Marolda R. Calissano P. (2008). NGF and BDNF signaling control amyloidogenic route and Aβ production in hippocampal neurons. Proc. Natl. Acad. Sci. U S A 105, 13139–13144. 10.1073/pnas.0806133105 18728191 PMC2525562

[B35] Mesulam M. (2004). The cholinergic lesion of Alzheimer’s disease: pivotal factor or side show? Learn. Mem. 11, 43–49. 10.1101/lm.69204 14747516

[B36] Nagahara A. H. Tuszynski M. H. (2011). Potential therapeutic uses of BDNF in neurological and psychiatric disorders. Nat. Rev. Drug Discov. 10, 209–219. 10.1038/nrd3366 21358740

[B37] Peng S. Wuu J. Mufson E. J. Fahnestock M. (2005). Precursor form of brain-derived neurotrophic factor and mature brain-derived neurotrophic factor are decreased in the pre-clinical stages of Alzheimer’s disease. J. Neurochem. 93, 1412–1421. 10.1111/j.1471-4159.2005.03135.x 15935057

[B38] Perez S. E. Dar S. Ikonomovic M. D. DeKosky S. T. Mufson E. J. (2007). Cholinergic forebrain degeneration in the APPswe/PS1ΔE9 transgenic mouse. Neurobiol. Dis. 28, 3–15. 10.1016/j.nbd.2007.06.015 17662610 PMC2245889

[B39] Phillips H. S. Hains J. M. Armanini M. Laramee G. R. Johnson S. A. Winslow J. W. (1991). BDNF mRNA is decreased in the hippocampus of individuals with Alzheimer’s disease. Neuron 7, 695–702. 10.1016/0896-6273(91)90273-3 1742020

[B40] Probst A. Götz J. Wiederhold K. H. Tolnay M. Mistl C. Jaton A. L.. (2000). Axonopathy and amyotrophy in mice transgenic for human four-repeat tau protein. Acta Neuropathol. 99, 469–481. 10.1007/s004010051148 10805089

[B41] Ramos-Rodriguez J. J. Pacheco-Herrero M. Thyssen D. Murillo-Carretero M. I. Berrocoso E. Spires-Jones T. L.. (2013). Rapid β-amyloid deposition and cognitive impairment after cholinergic denervation in APP/PS1 mice. J. Neuropathol. Exp. Neurol. 72, 272–285. 10.1097/NEN.0b013e318288a8dd 23481704 PMC3612835

[B42] Rantamäki T. Kemppainen S. Autio H. Stavén S. Koivisto H. Kojima M.. (2013). The impact of Bdnf gene deficiency to the memory impairment and brain pathology of APPswe/PS1dE9 mouse model of Alzheimer’s disease. PLoS One 8:e68722. 10.1371/journal.pone.0068722 23844236 PMC3700921

[B43] Schliebs R. Arendt T. (2011). The cholinergic system in aging and neuronal degeneration. Behav. Brain Res. 221, 555–563. 10.1016/j.bbr.2010.11.058 21145918

[B44] Schmitz T. W. Nathan Spreng R. Alzheimer’s Disease Neuroimaging Initiative. (2016). Basal forebrain degeneration precedes and predicts the cortical spread of Alzheimer’s pathology. Nat. Commun. 7:13249. 10.14264/uql.2015.328 27811848 PMC5097157

[B45] Siegel G. J. Chauhan N. B. (2000). Neurotrophic factors in Alzheimer’s and Parkinson’s disease brain. Brain Res. Rev. 33, 199–227. 10.1016/s0165-0173(00)00030-8 11011066

[B46] Tait D. S. Brown V. J. (2008). Lesions of the basal forebrain impair reversal learning but not shifting of attentional set in rats. Behav. Brain Res. 187, 100–108. 10.1016/j.bbr.2007.08.035 17920704

[B47] Tapia-Arancibia L. Aliaga E. Silhol M. Arancibia S. (2008). New insights into brain BDNF function in normal aging and Alzheimer disease. Brain Res. Rev. 59, 201–220. 10.1016/j.brainresrev.2008.07.007 18708092

[B48] Tempier A. He J. Zhu S. Zhang R. Kong L. Tan Q.. (2013). Quetiapine modulates conditioned anxiety and alternation behavior in Alzheimer’s transgenic mice. Curr. Alzheimer Res. 10, 199–206. 10.2174/1567205011310020010 22950914

[B49] Turnbull M. T. Coulson E. J. (2017). Cholinergic basal forebrain lesion decreases neurotrophin signaling without affecting tau hyperphosphorylation in genetically susceptible mice. J. Alzheimers Dis. 55, 1141–1154. 10.3233/jad-160805 27767994

[B50] Volianskis A. Køstner R. Mølgaard M. Hass S. Jensen M. S. (2010). Episodic memory deficits are not related to altered glutamatergic synaptic transmission and plasticity in the CA1 hippocampus of the APPswe/PS1δE9-deleted transgenic mice model of ss-amyloidosis. Neurobiol. Aging 31, 1173–1187. 10.1016/j.neurobiolaging.2008.08.005 18790549

[B51] Yamamoto-Sasaki M. Ozawa H. Saito T. Rösler M. Riederer P. (1999). Impaired phosphorylation of cyclic AMP response element binding protein in the hippocampus of dementia of the Alzheimer type. Brain Res. 824, 300–303. 10.1016/s0006-8993(99)01220-2 10196463

[B52] Yasutake C. Kuroda K. Yanagawa T. Okamura T. Yoneda H. (2006). Serum BDNF, TNF-α and IL-1β levels in dementia patients: comparison between Alzheimer’s disease and vascular dementia. Eur. Arch. Psychiatry Clin. Neurosci. 256, 402–406. 10.1007/s00406-006-0652-8 16783499

